# Beyond Morphology: Pretransplant Tumor-to-Liver FDG Uptake Ratio Predicts Hepatocellular Carcinoma Recurrence After Liver Transplantation

**DOI:** 10.3390/jcm15218611

**Published:** 2026-11-05

**Authors:** Ahmet Atasever, Sinan Efe Yazici, Yildiray Yuzer

**Affiliations:** 1 Department of Surgery, Bahçelievler Medipol University Hospital, Istanbul Medipol University, Istanbul 34197, Türkiye; 2 Department of Surgery, Faculty of Medicine, Hacettepe University, Ankara 06100, Türkiye; sinanefeyazici@gmail.com; 3 Department of Liver Transplantation, Istanbul Florence Nightingale Hospital, Istanbul 34381, Türkiye; yildirayyuzer@gmail.com

**Keywords:** hepatocellular carcinoma, liver transplantation, FDG PET/CT, standardized uptake value, tumor-to-liver ratio, recurrence, Milan criteria

## Abstract

**Background/Objectives**: Tumor size and number do not fully capture the risk of hepatocellular carcinoma (HCC) recurrence after liver transplantation. We examined whether the pretransplant tumor-to-liver maximum standardized uptake value (SUV) ratio on ^18^F-FDG PET/CT is associated with recurrence, and assessed the previously reported cutoff of 1.43 in an independent cohort. **Methods**: We retrospectively analyzed 227 adults who underwent liver transplantation for HCC between July 2004 and November 2024 and had pretransplant FDG PET/CT. The primary endpoint was time to recurrence (TTR). The SUV ratio was analyzed as a continuous, log_2_-transformed variable in Cox models adjusted for age, MELD-Na, alpha-fetoprotein (AFP), and Milan status; the 1.43 cutoff was a secondary analysis. Death without recurrence was treated as a competing event. **Results**: Over a median follow-up of 80 months, 43 patients developed recurrence and 86 died. Each doubling of the SUV ratio was associated with a higher recurrence rate (adjusted HR 6.68; 95% CI 3.58–12.47) and higher mortality (HR 2.79; 95% CI 1.72–4.53). The 5-year cumulative incidence of recurrence was 44.7% with a ratio >1.43 and 7.5% with a ratio ≤1.43. Only 2 of 51 non-FDG-avid tumors recurred. The ratio added prognostic information to Milan criteria and AFP (likelihood ratio χ^2^ 28.5; *p* < 0.001) and stratified risk within the Milan criteria (5-year cumulative incidence of recurrence, 26.4% vs. 5.2%). **Conclusions**: A high pretransplant tumor-to-liver SUV ratio was strongly associated with recurrence independently of Milan status and AFP. External validation with standardized PET protocols is needed before clinical use.

## 1. Introduction

When hepatocellular carcinoma (HCC) arises in a cirrhotic liver, treatment depends on both tumor characteristics and the liver’s functional reserve. Liver transplantation treats the tumor and the underlying liver disease simultaneously [1,2]. However, recurrence after transplantation remains the main factor limiting its benefit, which is why the tumor’s biological behavior matters as much as its size when candidates are selected.

The Milan criteria, based on tumor size and number, became the foundation for candidate selection [3]. The UCSF criteria and subsequent approaches showed that morphological limits can be expanded in carefully selected patients [4,5]. However, tumors of similar size do not carry the same risk of recurrence. Current selection strategies therefore also consider alpha-fetoprotein (AFP), response to locoregional therapy, and other markers of tumor biology [6,7]. The Metroticket 2.0 model combines AFP with tumor burden, whereas the RETREAT score uses AFP at transplantation together with explant findings [8,9]. Because RETREAT depends on pathology, it cannot be used for selection before transplantation.

^18^F-fluorodeoxyglucose positron emission tomography (FDG PET) reflects tumor glucose metabolism, and several studies in transplant recipients have linked FDG uptake to recurrence [10,11]. Normalizing tumor uptake to liver parenchymal uptake accounts for patient-specific background activity. However, studies use different reference regions and cutoffs [12,13].

Zheng et al. used a tumor SUVmax-to-liver SUVmax ratio cutoff of 1.43 and found that values above this threshold were associated with recurrence after transplantation [14]. In this study, we evaluated the association between the SUV ratio and recurrence as a continuous measure among patients transplanted at our center, assessed the 1.43 cutoff in an independent cohort, and determined whether the ratio provides additional information beyond established selection criteria.

## 2. Materials and Methods

### 2.1. Study Design, Setting, and Ethics

This retrospective cohort study reviewed the records of adult patients who underwent liver transplantation at the Liver Transplantation Center of Demiroğlu Bilim University Florence Nightingale Hospital between July 2004 and November 2024. The study was approved by the Medical Research Ethics Committee of Demiroğlu Bilim University (decision no. 51016662/43342; 27 November 2024). As part of the routine institutional transplant consent process, written consent permitting the use of clinical data for scientific research was obtained from all patients before transplantation. The retrospective use of these data for the present study was approved by the institutional ethics committee. Reporting follows the STROBE guideline [15].

### 2.2. Patients

**Figure 1:**
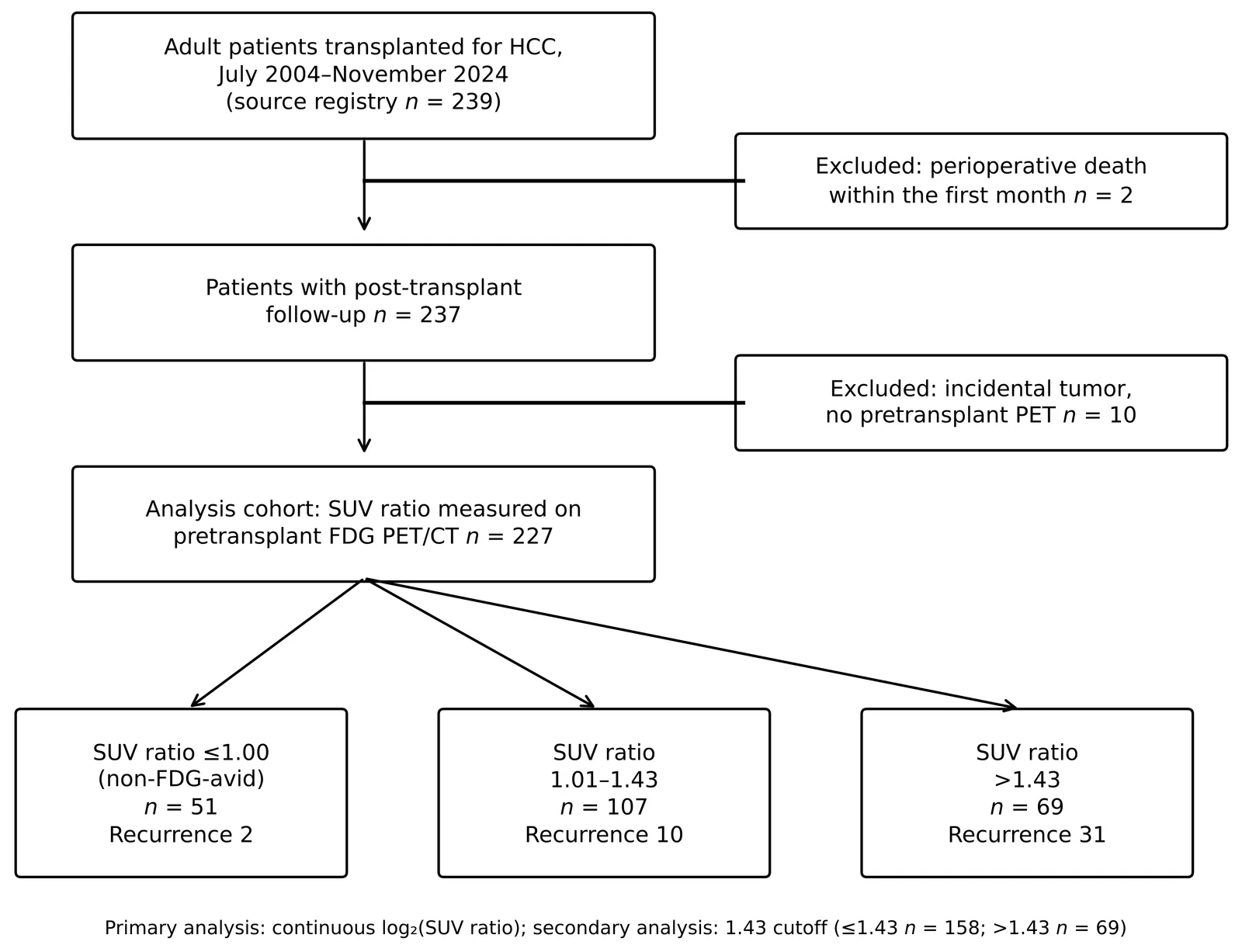
Patient flow diagram.

The source registry included 239 adult patients with HCC who underwent liver transplantation between July 2004 and November 2024. Two patients who died from non-oncologic perioperative causes within the first month after transplantation were excluded from the analysis. Among the remaining 237 patients, 10 had incidental HCC detected only on explant pathology and therefore lacked a pretransplant FDG PET/CT examination. These patients were excluded from the PET-based analysis. The final study cohort consisted of 227 patients with available pretransplant tumor-to-liver SUV ratio measurements (Figure 1).

Twenty-four patients with a cholangiocarcinoma component on explant pathology (combined HCC–cholangiocarcinoma) were retained in the primary analysis cohort, because this component was identified only on explant pathology and was not known at the time of pretransplant evaluation. To assess the robustness of the findings, a sensitivity analysis excluding these patients was also performed. No formal sample-size calculation was performed; the sample size was determined by all patients who met the eligibility criteria and had complete data during the study period.

### 2.3. Clinical Variables

Age, sex, etiology of cirrhosis, Child–Pugh class, and MELD-Na score at the time of transplantation were recorded. Donor type was classified as living or deceased. Milan and UCSF status were determined from the most recent contrast-enhanced computed tomography (CT) or magnetic resonance imaging (MRI) performed before transplantation. Serum alpha-fetoprotein (AFP) was defined as the last available pretransplant measurement and was expressed in ng/mL. For descriptive analyses, AFP was categorized as <10, 10–199, 200–399, and ≥400 ng/mL; in regression models, it was analyzed as log_2_(AFP + 1).

Before transplantation, some patients received locoregional therapy with transarterial chemoembolization, transarterial radioembolization, radiofrequency ablation, or microwave ablation for bridging or downstaging. The use of any pretransplant locoregional therapy was recorded as a binary variable.

### 2.4. FDG PET/CT Imaging

At our center, FDG PET/CT is routinely performed during the pretransplant evaluation of all liver transplant candidates with known or suspected HCC, primarily to exclude extrahepatic disease. Accordingly, all patients with a pretransplant diagnosis of HCC in the study period underwent FDG PET/CT; only the 10 patients with incidental HCC on explant pathology did not. According to our institutional protocol, FDG PET/CT was performed as part of the final pretransplant evaluation, within 1 month before transplantation. All PET/CT examinations were performed at our institution; however, the PET/CT scanners were replaced during the study period. In patients who received locoregional therapy, for either bridging or downstaging, PET/CT was performed at least 4 weeks after the last treatment and therefore reflected the metabolic activity of residual viable tumor tissue.

Patients fasted for at least 6 h before imaging, and blood glucose levels were confirmed to be below 200 mg/dL before FDG administration. FDG was administered intravenously at a weight-adjusted dose, followed by an uptake period of approximately 60 min in a quiet room. Images were acquired from the skull base to the mid-thigh. Low-dose CT was used for attenuation correction and anatomical localization. PET images were reconstructed using an iterative algorithm, and standardized uptake values were normalized to body weight in accordance with international procedure guidelines [16].

### 2.5. Measurement of the SUV Ratio

PET/CT fusion images were reviewed together with the corresponding pretransplant CT or MRI. For tumor assessment, a three-dimensional volume of interest (VOI) encompassing the entire lesion with the highest FDG uptake was drawn, and the tumor SUVmax was recorded. In patients with multiple tumors, the lesion showing the highest FDG uptake was selected. For the liver reference, an approximately 3 cm-diameter spherical VOI was placed in normal-appearing right hepatic lobe parenchyma, avoiding the tumor, major vessels, bile ducts, and previously treated areas, and the liver SUVmax was recorded. The tumor-to-liver SUV ratio was calculated by dividing tumor SUVmax by liver SUVmax.

SUV measurements were performed retrospectively for this study by two experienced liver transplant surgeons, who reviewed the PET/CT images together with the corresponding CT or MRI and reached a consensus using the VOI criteria described above. This approach was used because liver reference values were not consistently available in the original PET/CT reports. Outcome data were not reviewed during the measurements; however, because the readers were members of the transplant team, they may have been aware of the clinical course of some patients.

### 2.6. Explant Pathology

The number of viable tumors, largest tumor diameter, total tumor diameter, histological grade, microvascular invasion, macrovascular invasion, and the presence of a cholangiocarcinoma component were recorded from the explant pathology. Among patients who had received pretransplant locoregional therapy, the absence of viable tumor on explant was considered a complete pathological response. In these patients, tumor diameter, number, and histological grade could not be assessed and were reported as a separate category. Milan status was reassessed using explant findings, defined as a single tumor ≤5 cm or up to three tumors each ≤3 cm, without macrovascular invasion. Patients who were within Milan criteria before transplantation but outside the criteria on explant pathology were classified as having been understaged preoperatively. The RETREAT score was calculated from explant findings [9].

### 2.7. Outcomes

The primary endpoint was time to recurrence (TTR), defined as the time from liver transplantation to the first documented HCC recurrence. Patients who died without recurrence were censored at the date of death, and patients who remained alive and recurrence-free were censored at their last follow-up. Secondary endpoints were overall survival (OS), defined as death from any cause, and recurrence-free survival (RFS), defined as the time to either HCC recurrence or death, whichever occurred first.

HCC recurrence was diagnosed primarily on the basis of new lesions consistent with HCC on contrast-enhanced CT or MRI, with serum AFP levels and histological confirmation by biopsy used as supportive evidence when clinically indicated. The date of recurrence was defined as the date on which recurrence was first documented. Post-transplant surveillance with serum AFP and contrast-enhanced CT or MRI was performed according to routine clinical practice rather than a predefined surveillance schedule. Follow-up was based on visits at our center; patients who continued follow-up at other institutions were censored at their last visit at our center.

### 2.8. Use of the Cutoff

In the primary analysis, the SUV ratio was analyzed as a continuous variable. Because of its right-skewed distribution, the ratio was log_2_-transformed; therefore, the corresponding hazard ratio represents the change in hazard associated with a twofold increase in the SUV ratio. In secondary analyses, patients were classified according to the previously reported cutoff of 1.43 described by Zheng et al. [14]. The 1.43 threshold was treated as an externally reported cutoff rather than a data-derived threshold in the present cohort. Because dichotomization of a continuous predictor may result in loss of information and may exaggerate effect estimates [17], the continuous SUV ratio was retained as the primary exposure. To examine whether the findings depended on a specific threshold, sensitivity analyses were also performed using cutoffs ranging from 1.00 to 2.00.

### 2.9. Statistical Analysis

Categorical variables were summarized as numbers and percentages, and continuous variables as medians with interquartile ranges (IQRs). Comparisons across SUV ratio groups were performed using the chi-square test for categorical variables and the Kruskal–Wallis test for continuous variables. Median follow-up was estimated using the reverse Kaplan–Meier method.

Time to recurrence (TTR) and overall survival (OS) were estimated using the Kaplan–Meier method, and groups were compared using the log-rank test. To account for the competing risk of death without recurrence, the cumulative incidence of recurrence was estimated using the Aalen–Johansen estimator and compared between groups using Gray’s test [18]. In an additional competing-risk analysis, Fine–Gray subdistribution hazard models were fitted using the same covariates as the primary adjusted model. Recurrence probabilities according to clinical characteristics were estimated as cumulative incidences with death without recurrence as a competing event, and groups were compared using Gray’s test.

Associations with TTR and OS were evaluated using univariable and multivariable Cox proportional hazards regression. The primary adjusted model included the SUV ratio, age per 10-year increase, MELD-Na per 5-point increase, log_2_(AFP + 1), and pretransplant Milan status. These covariates were selected because they were available before transplantation and represented clinically established prognostic factors, while the number of covariates was limited in relation to the number of observed events to reduce the risk of overfitting [19]. Explant-derived variables were not included in the primary model because they were unavailable at the time of pretransplant assessment. Tied event times were handled using the Breslow method. The proportional hazards assumption was assessed using the Grambsch–Therneau test based on scaled Schoenfeld residuals. The functional form of the continuous SUV ratio was assessed by modelling log_2_(SUV ratio) with a natural cubic spline with three degrees of freedom (knots at the tertiles) in the primary adjusted model and comparing this model with the linear model using a likelihood-ratio test.

Recurrence-free survival was evaluated in a secondary Cox proportional hazards analysis using the same covariates as the primary TTR model: log_2_-transformed SUV ratio, age, MELD-Na, log_2_(AFP + 1), and pretransplant Milan status. Patients who were alive without recurrence were censored at their last follow-up.

The incremental prognostic value of the SUV ratio beyond established criteria was assessed using likelihood-ratio tests comparing nested Cox models. Comparisons were performed against models based on pretransplant Milan criteria, UCSF criteria, Milan criteria combined with AFP, and the explant-based RETREAT score. The association between the SUV ratio and recurrence was also examined in clinically relevant subgroups, including patients within and beyond the Milan criteria, patients with AFP <200 ng/mL, and patients within the Milan criteria on explant pathology.

To explore the biological correlates of the SUV ratio, associations with microvascular invasion, macrovascular invasion, and histological grade 3 were evaluated using logistic regression. We also assessed the change in the hazard ratio for the SUV ratio after these explant-derived variables were added to the primary Cox model.

Sensitivity analyses were performed by separately excluding patients with an SUV ratio of exactly 1.00, patients with non-FDG-avid tumors (SUV ratio ≤ 1.00), patients with a cholangiocarcinoma component, patients with macrovascular invasion, patients without viable tumor on explant pathology, and patients who had received pretransplant bridging therapy. In an additional analysis, bridging therapy was included as a covariate in the primary model. Similarly, the cholangiocarcinoma component was added as a covariate, and its interaction with the SUV ratio was tested.

The ability of the SUV ratio to discriminate recurrence at 3 and 5 years was assessed using horizon-specific ROC analyses. Cases were defined as patients who developed recurrence within the specified time horizon, whereas controls were patients who remained recurrence-free beyond that time or died without recurrence before that time. Patients who were alive and recurrence-free but were lost to follow-up before the specified time horizon were excluded from these analyses. Areas under the curve (AUCs) were reported with 95% confidence intervals (CIs) calculated using the DeLong method, and sensitivity and specificity were estimated for the externally reported cutoff of 1.43. To quantify incremental discrimination, horizon-specific AUCs at 3 and 5 years were compared between a Cox model including pretransplant Milan status and log_2_(AFP + 1) and the same model with log_2_(SUV ratio) added, using the same case and control definitions. The 95% CI for the difference in AUC was obtained from 2000 bootstrap samples, with both models refitted in each sample.

All statistical tests were two-sided, and *p* < 0.05 was considered statistically significant. Analyses were performed using R version 4.6.1 (R Foundation for Statistical Computing, Vienna, Austria), with the survival package (version 3.8-12) and cmprsk package (version 2.2-12).

## 3. Results

### 3.1. Patient Characteristics

The analysis cohort comprised 227 patients (Figure 1). The median age was 57 years (IQR, 53–62), and 85.5% were men. Living donor liver transplantation was performed in 194 patients (85.5%). The median tumor-to-liver SUV ratio was 1.22 (IQR, 1.03–1.50). Fifty-one patients (22.5%) had non-FDG-avid tumors, 107 (47.1%) had an SUV ratio of 1.01–1.43, and 69 (30.4%) had an SUV ratio >1.43 (Table 1).

Patients with higher SUV ratios were more frequently beyond the Milan and UCSF criteria before transplantation and had higher AFP levels. On explant pathology, higher SUV ratios were also associated with larger tumors and higher frequencies of microvascular invasion, macrovascular invasion, and grade 3 histology. Microvascular invasion was present in 49.3%, 23.4%, and 9.8% of patients in the >1.43, 1.01–1.43, and ≤1.00 groups, respectively. Age, sex, cirrhosis etiology, Child–Pugh class, MELD-Na score, use of bridging therapy, and donor type were similar across groups (Table 1).

Among the 136 patients who were within the Milan criteria before transplantation, 37 (27.2%) were beyond the criteria on explant pathology. This proportion was 38.7% (12/31) among patients with an SUV ratio >1.43 and 23.8% (25/105) among those with a ratio ≤1.43 (*p* = 0.159). Of the 91 patients who were beyond the Milan criteria before transplantation, 22 were within the criteria on explant pathology.

### 3.2. Recurrence and Survival

The median follow-up was 80 months, as estimated by the reverse Kaplan–Meier method. During follow-up, 43 patients (18.9%) developed HCC recurrence and 86 (37.9%) died; 46 patients died without recurrence.

**Figure 2:**
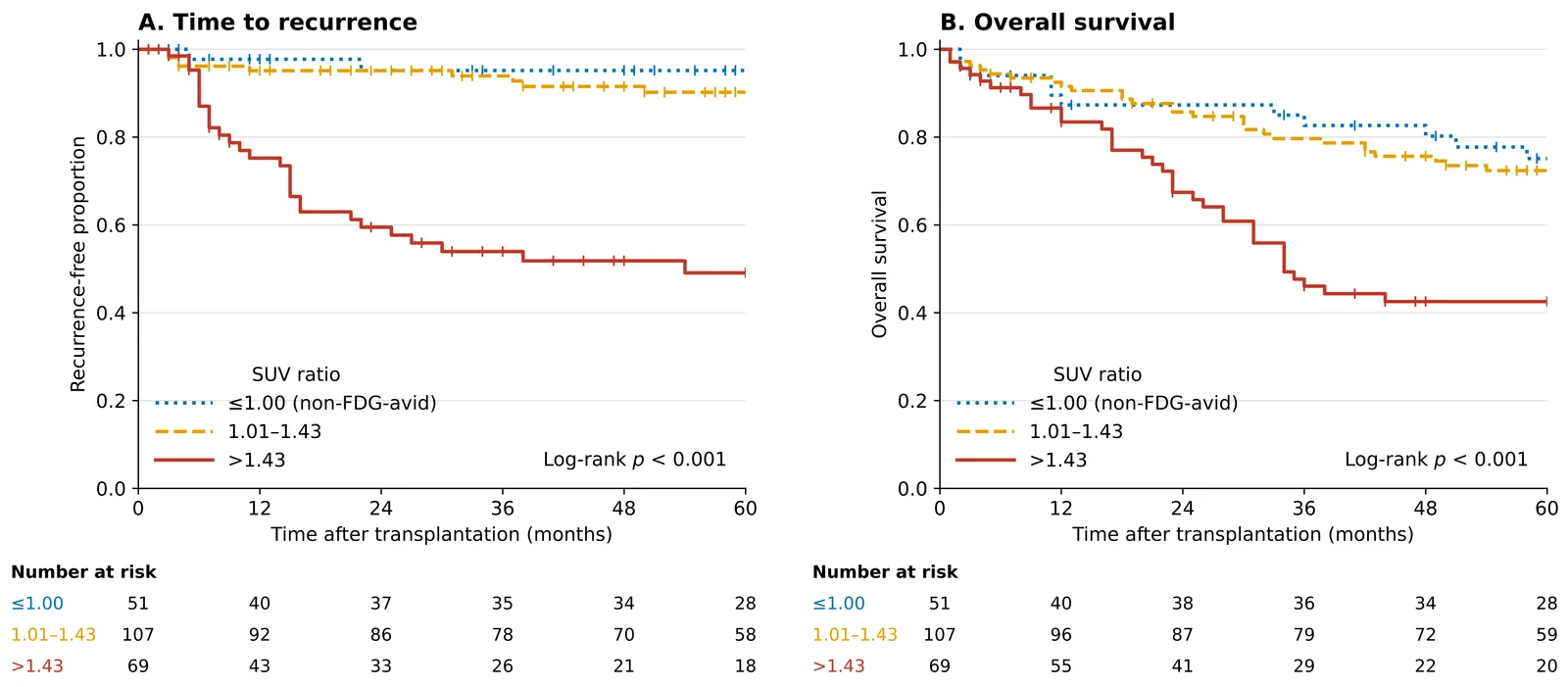
Kaplan–Meier estimates by SUV ratio group: (**A**) time to recurrence, with patients who died without recurrence censored at death; (**B**) overall survival. Curves are truncated at 60 months; *p* values were calculated using the entire follow-up period.

**Figure 3:**
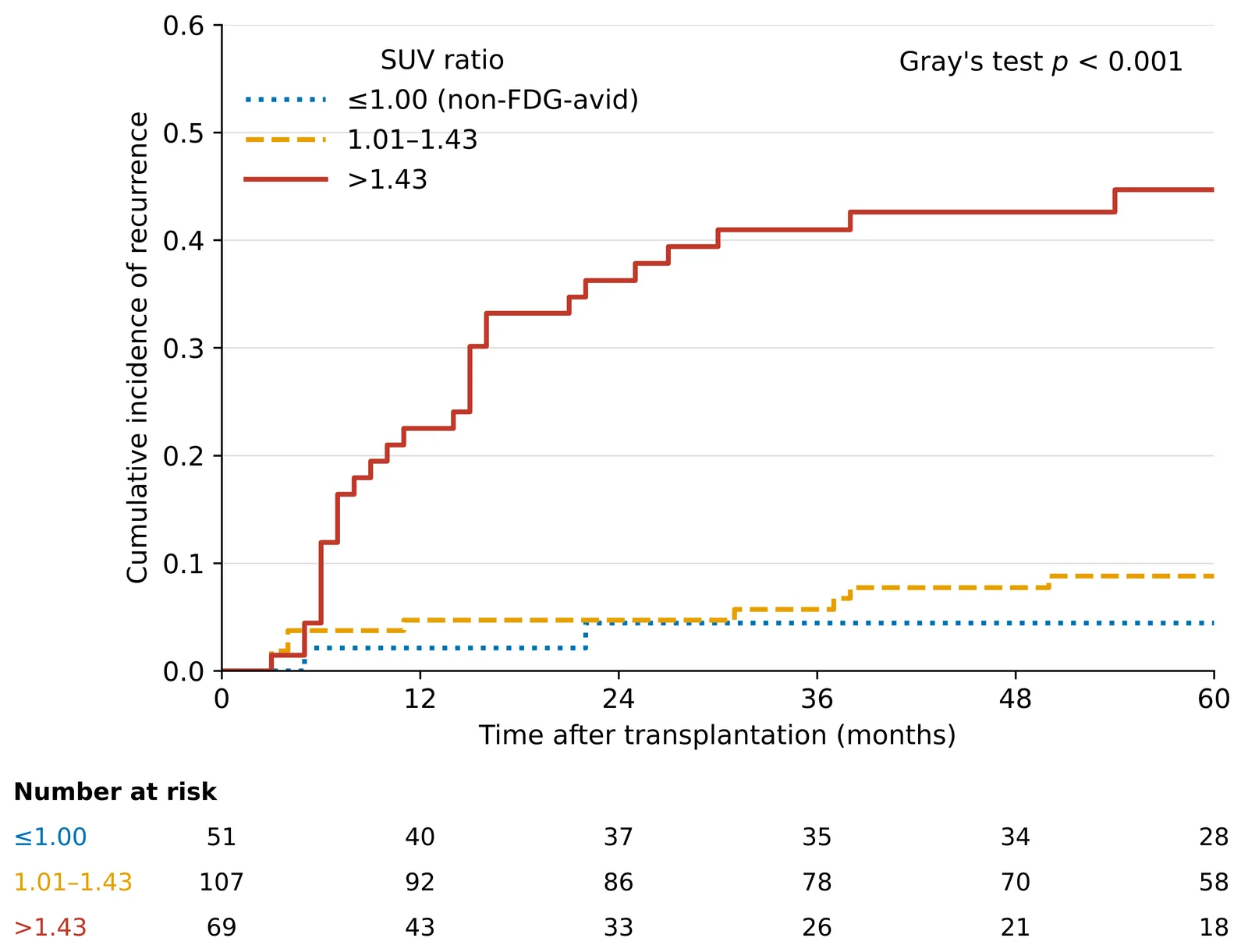
Cumulative incidence of recurrence by SUV ratio group, with death without recurrence treated as a competing event (Aalen–Johansen estimator; Gray’s test *p* < 0.001). Curves are truncated at 60 months; *p* values were calculated using the entire follow-up period.

Recurrence occurred in 2 of 51 patients (3.9%) in the non-FDG-avid group, 10 of 107 (9.3%) in the 1.01–1.43 group, and 31 of 69 (44.9%) in the >1.43 group. TTR differed significantly across the three groups (log-rank *p* < 0.001; Figure 2A). When death without recurrence was treated as a competing event, the 5-year cumulative incidence of recurrence was 4.4%, 8.8%, and 44.7%, respectively (Gray’s test *p* < 0.001; Figure 3). Five-year OS was 75.1%, 72.4%, and 42.6%, respectively (log-rank *p* < 0.001; Figure 2B). Cumulative incidence estimates and adjusted Fine–Gray analyses are presented in Table S1. Each doubling of the SUV ratio was associated with a higher subdistribution hazard of recurrence (adjusted SHR, 5.84; 95% CI, 3.31–10.29; *p* < 0.001). The association was also observed for an SUV ratio >1.43 (adjusted SHR, 6.74; 95% CI, 3.41–13.32; *p* < 0.001).

In adjusted analyses, the 1.01–1.43 and non-FDG-avid groups did not differ significantly with respect to TTR (HR, 2.07; 95% CI, 0.45–9.51; *p* = 0.350) or OS (HR, 1.06; 95% CI, 0.56–2.01; *p* = 0.851). Estimated recurrence probabilities according to clinical characteristics are presented in Table 2.

### 3.3. Primary Analysis: Continuous SUV Ratio

In the adjusted primary model, each doubling of the SUV ratio was associated with a higher hazard of recurrence (HR, 6.68; 95% CI, 3.58–12.47; *p* < 0.001). In the same model, log_2_(AFP + 1) (HR, 1.14; 95% CI, 1.01–1.27; *p* = 0.030) and being beyond the Milan criteria (HR, 2.19; 95% CI, 1.10–4.35; *p* = 0.025) were also associated with recurrence. The SUV ratio was also associated with overall mortality (HR, 2.79; 95% CI, 1.72–4.53; *p* < 0.001) (Table 3). In the secondary adjusted analysis, each doubling of the SUV ratio was associated with a higher hazard of recurrence or death (HR, 2.92; 95% CI, 1.83–4.65; *p* < 0.001; Table S7). There was some evidence of departure from log-linearity for recurrence (*p* = 0.030) but not for overall mortality (*p* = 0.34). The spline showed little change in hazard up to a ratio of approximately 1.1, a steep increase between approximately 1.1 and 1.7, and a flattening at higher values (Figure S1). The hazard ratio per doubling should therefore be interpreted as an average summary of this relationship.

### 3.4. Secondary Analysis: The 1.43 Cutoff

For patients with an SUV ratio >1.43, the adjusted HR was 7.08 (95% CI 3.53–14.18; *p* < 0.001) for recurrence and 2.76 (95% CI 1.76–4.34; *p* < 0.001) for OS. The 5-year cumulative incidence of recurrence was 7.5% (95% CI 3.9–12.5) with a ratio ≤1.43 and 44.7% (95% CI 32.1–56.5) with a ratio >1.43 (Gray’s test *p* < 0.001). The association persisted in the Fine–Gray model, with a subdistribution HR of 5.84 (95% CI 3.31–10.29) for the continuous ratio and 6.74 (95% CI 3.41–13.32) for a ratio >1.43 (both *p* < 0.001; Table S1). The association remained significant at every cutoff between 1.10 and 2.00 (adjusted HR range 4.05–7.08; Table S2). For histological grade 3, the reference group includes the four patients without viable tumor.

### 3.5. Incremental Value over Existing Criteria

Adding the SUV ratio significantly improved Cox model fit compared with models based on Milan criteria, UCSF criteria, Milan criteria plus AFP, and the base clinical model. The ratio also added significant information to the explant-based RETREAT score (χ^2^ 22.2; *p* < 0.001). By contrast, with the SUV ratio already in the model, the combined contribution of Milan status and AFP was smaller (χ^2^ 12.5; *p* = 0.002) (Table S3).

Among the 136 patients within the pretransplant Milan criteria, the 5-year cumulative incidence of recurrence was 26.4% with an SUV ratio >1.43 and 5.2% with a ratio ≤1.43 (Gray’s test *p* < 0.001; HR, 7.61; 95% CI, 2.67–21.66; *p* < 0.001). The association was also present in patients beyond Milan criteria (HR 6.78; 95% CI 2.74–16.81), in those with AFP <200 ng/mL (HR 7.23; 95% CI 3.48–15.02), and in those within Milan criteria on explant (HR 6.12; 95% CI 2.04–18.32) (Table S4).

### 3.6. SUV Ratio and Explant Findings

Each doubling of the SUV ratio was associated with higher odds of microvascular invasion (OR, 4.54; 95% CI, 2.20–9.38), macrovascular invasion (OR, 3.08; 95% CI, 1.41–6.70), and histological grade 3 (OR, 3.02; 95% CI, 1.53–5.96). These associations remained after adjustment for AFP and pretransplant Milan status. When microvascular invasion, macrovascular invasion, and histological grade 3 were added to the primary adjusted Cox model, the HR for the SUV ratio decreased from 6.68 to 5.79 (95% CI, 2.95–11.36) but remained statistically significant (Table S5).

### 3.7. Discrimination of Recurrence

**Figure 4:**
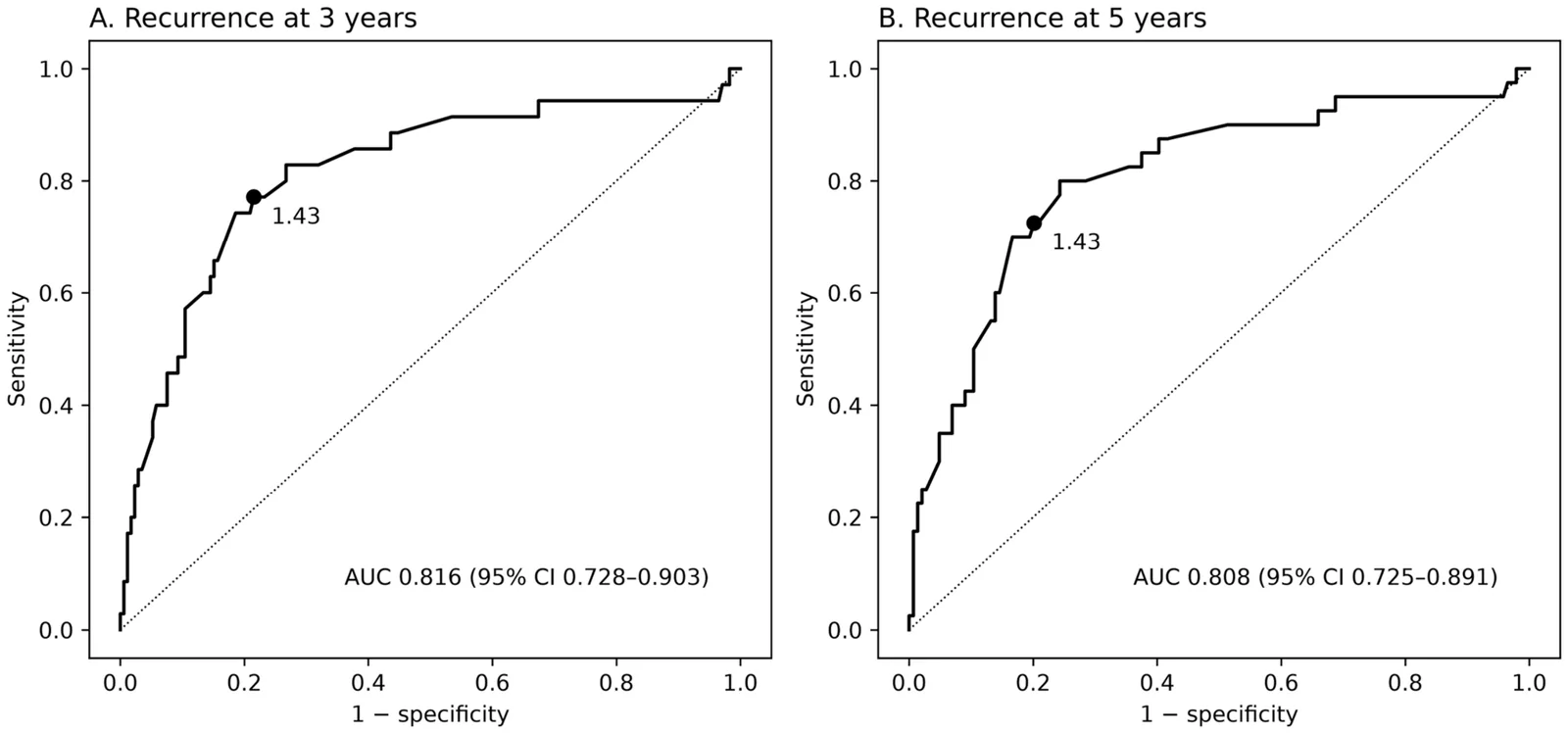
Receiver operating characteristic curves of the SUV ratio for recurrence at 3 years (**A**) and 5 years (**B**). The dot marks the 1.43 cutoff. The dot marks the 1.43 cutoff, and the diagonal dotted line represents no discrimination (AUC = 0.5).

The SUV ratio showed good discrimination for recurrence at both 3 and 5 years. The AUC was 0.816 (95% CI, 0.728–0.903) at 3 years and 0.808 (95% CI, 0.725–0.891) at 5 years (Figure 4). At the externally reported cutoff of 1.43, sensitivity and specificity were 77.1% and 78.5%, respectively, at 3 years and 72.5% and 79.9%, respectively, at 5 years. Adding the SUV ratio to a model including Milan status and AFP increased the AUC from 0.784 to 0.856 at 3 years (difference, 0.072; 95% CI, 0.009–0.154) and from 0.752 to 0.835 at 5 years (difference, 0.082; 95% CI, 0.012–0.164).

### 3.8. Sensitivity Analyses

In exploratory analyses using alternative SUV ratio cutoffs from 1.00 to 2.00, the association with TTR remained statistically significant across all evaluated thresholds (Table S2). After excluding the 40 patients with an SUV ratio of exactly 1.00, the adjusted HR for recurrence was 5.42 (95% CI, 2.80–10.48) for the continuous SUV ratio and 5.43 (95% CI, 2.72–10.84) for the 1.43 cutoff. The association between the SUV ratio and recurrence remained significant after separately excluding patients with non-FDG-avid tumors, a cholangiocarcinoma component, macrovascular invasion, no viable tumor on explant pathology, or prior bridging therapy. The findings were also unchanged when bridging therapy was added as a covariate to the primary model (Table S6). Adding the cholangiocarcinoma component to the primary model did not materially change the association (HR per doubling, 6.95; 95% CI, 3.69–13.10), and there was no significant interaction between the SUV ratio and the cholangiocarcinoma component (*p* = 0.43; Table S6). There was no evidence of violation of the proportional hazards assumption for the SUV ratio in the TTR and OS models (*p* = 0.14 and *p* = 0.23, respectively).

## 4. Discussion

In this cohort, the pretransplant tumor-to-liver SUV ratio showed a strong, graded association with HCC recurrence after liver transplantation. This association remained after adjustment for age, MELD-Na, AFP, and Milan status, both when the SUV ratio was analyzed as a continuous variable and when the previously reported cutoff of 1.43 was applied. The SUV ratio provided additional prognostic information beyond the Milan and UCSF criteria and AFP, and identified a subgroup with a higher risk of recurrence even among patients within the Milan criteria.

Tumor size and number alone do not fully capture the risk of recurrence after transplantation. The Milan and UCSF criteria describe morphological tumor burden, whereas more recent models recognize that biological risk may vary even among patients with a similar tumor burden [3,4,6]. In our primary adjusted model, Milan status and AFP remained associated with recurrence, suggesting that the SUV ratio complements rather than replaces these established factors. Among patients within the pretransplant Milan criteria, the 5-year cumulative incidence of recurrence was higher with an SUV ratio >1.43 than with a ratio ≤1.43 (26.4% vs. 5.2%). Although these patients would generally be considered at lower risk on the basis of morphological criteria, metabolic assessment may help identify a subgroup that warrants closer post-transplant surveillance. The role of AFP in the pretransplant assessment of tumor biology is also well established [20].

Lee et al. reported an association between FDG PET findings and recurrence after transplantation, and a later study in living donor recipients linked PET positivity particularly to early recurrence [10,11]. Kornberg et al. found that increased FDG uptake was associated with microvascular invasion [21], and more recent data have also linked tumor-to-liver FDG uptake ratios with microvascular invasion [22]. In our study, the SUV ratio was associated with microvascular invasion, macrovascular invasion, and grade 3 histology. When these explant findings were added to the model, the association between the SUV ratio and recurrence was only modestly attenuated, suggesting that the ratio may capture biological information beyond vascular invasion and tumor differentiation. Previous studies have linked increased FDG uptake in HCC to the expression of pSTAT3, HIF-1α, and GLUT1, as well as to immune and vascular characteristics of the tumor [23,24]. Because molecular and immunohistochemical markers such as pSTAT3, HIF-1α, GLUT1, PD-L1, and tumor immune and vascular features were not assessed in our cohort, the biological mechanisms underlying these associations cannot be determined from our data.

The recurrence rate among patients with non-FDG-avid tumors (SUV ratio ≤ 1.00) was very low, with only 2 recurrences among 51 patients. This finding is consistent with previous studies linking absent or low FDG uptake with better tumor differentiation and less aggressive biological behavior [10,11,25]. However, recurrence and survival did not differ significantly between the non-FDG-avid and 1.01–1.43 groups, whereas recurrence risk was substantially higher in patients with an SUV ratio >1.43. These findings support considering non-FDG-avid tumors as part of the lower-SUV-ratio group rather than as a distinct prognostic category. The association between the SUV ratio and recurrence remained significant after patients with non-FDG-avid tumors were excluded.

Zheng et al. evaluated a tumor SUVmax-to-liver SUVmax ratio cutoff of 1.43 in derivation and temporal validation cohorts and reported an independent association with recurrence after transplantation [14]. In our cohort, an SUV ratio >1.43 was associated with recurrence after adjustment for age, MELD-Na, AFP, and Milan status (HR, 7.08; 95% CI, 3.53–14.18). Differences in patient selection, donor type, locoregional treatment, and PET acquisition protocols may limit direct comparisons between studies. Because the tumor and the reference liver are measured on the same scan under identical acquisition and reconstruction conditions, the SUV ratio is expected to be less sensitive to scanner-and reconstruction-related differences than tumor SUVmax alone. However, SUVmax is susceptible to image noise and partial-volume effects, and the 1.43 cutoff may therefore not be directly transferable across scanners and protocols. Validation of the SUV ratio in multicenter cohorts using different PET/CT systems is therefore needed. In our cohort, the association was also observed when the SUV ratio was analyzed continuously and across alternative cutoffs. These findings support further multicenter evaluation using standardized PET protocols before the 1.43 cutoff is adopted for clinical decision-making.

The 3- and 5-year AUCs indicate that the SUV ratio had good discriminatory ability for recurrence in this cohort. The SUV ratio also improved discrimination when added to Milan status and AFP, although these AUCs were estimated in the same cohort used to fit the models and may therefore be optimistic. However, discrimination at the group level does not necessarily translate into accurate prediction for individual patients; more than half of the patients with an SUV ratio >1.43 did not develop recurrence. Therefore, the SUV ratio alone should not be used to exclude patients from transplantation. Rather, it may help identify patients who could benefit from closer post-transplant surveillance and may be useful as a component of multivariable risk models. In practical terms, the SUV ratio is obtained before transplantation from an examination that is routinely performed in transplant candidates with known or suspected HCC at our center, and it can be considered alongside Milan status and AFP. A high ratio, particularly in a patient within the Milan criteria, may support closer post-transplant surveillance. In living donor liver transplantation, which accounted for most transplants in this cohort, pretransplant information on recurrence risk may also contribute to the risk–benefit discussion with the recipient and the donor. However, the 1.43 cutoff was derived in a different population and has now been assessed in only one additional center; it should therefore be validated in external, multicenter cohorts before it is used to guide candidate selection.

On explant pathology, 27.2% of patients who were within the Milan criteria before transplantation were found to be beyond the criteria. This proportion falls within the approximately 22.0–33.3% range reported in previous studies of patients classified as within the Milan criteria on pretransplant imaging but found to be beyond these criteria on explant pathology [26,27,28]. The proportion was numerically higher among patients with an SUV ratio >1.43, although the difference was not statistically significant.

This study has several limitations. First, it was retrospective and conducted at a single center, and patient selection and bridging strategies may have changed over the long study period; in addition, PET/CT scanners were replaced during this period, and the exact intervals between locoregional therapy, PET/CT, and transplantation could not be retrieved for all patients. Second, PET measurements were performed retrospectively by two liver transplant surgeons rather than a nuclear medicine physician, and although outcome data were not reviewed during the measurements, the readers may have been aware of the clinical course of some patients. Although SUVmax is a software-derived value and the VOIs were placed according to predefined criteria, measurement bias cannot be fully excluded. Because the measurements were obtained by consensus, interobserver agreement could not be assessed. Third, volume-based metabolic parameters, including metabolic tumor volume (MTV) and total lesion glycolysis (TLG), were not evaluated [29]. Fourth, post-transplant surveillance was based on routine clinical practice rather than a predefined schedule; therefore, recurrence may have been detected at different intervals across patients. Fifth, 24 patients with a combined HCC–cholangiocarcinoma component, which is associated with a more aggressive course, were retained in the primary cohort because this component was identified only on explant pathology; however, excluding these patients or adjusting for this component did not materially alter the findings. Finally, the results have not yet been validated in an external cohort.

## 5. Conclusions

The pretransplant tumor-to-liver SUV ratio on FDG PET/CT was associated with HCC recurrence after liver transplantation after adjustment for Milan status and AFP, and provided additional prognostic information beyond these established factors. The SUV ratio may help identify patients, particularly those within the Milan criteria, who could benefit from closer post-transplant surveillance. Standardization of the measurement protocol and external validation in multicenter cohorts are needed before the SUV ratio and the 1.43 cutoff can be incorporated into clinical practice.

## Figures and Tables

**Table 1:** Patient characteristics by SUV ratio group.

Variable	Level	All	≤1.00 (Non-FDG-Avid)	1.01–1.43	>1.43	*p*
Number of patients		227	51	107	69	
Age, years	median (IQR)	57.0 (53.0–62.0)	58.0 (51.5–64.0)	57.0 (53.0–62.0)	58.0 (53.0–62.0)	0.905
MELD-Na	median (IQR)	12.0 (9.0–16.0)	12.0 (9.0–16.5)	12.0 (9.0–16.0)	12.0 (9.0–17.0)	0.638
AFP, ng/mL	median (IQR)	13.5 (4.8–50.4)	7.3 (3.4–25.2)	12.6 (5.8–41.4)	21.1 (6.3–118.3)	0.004
SUV ratio	median (IQR)	1.22 (1.03–1.50)	1.00 (1.00–1.00)	1.20 (1.10–1.30)	1.71 (1.54–2.05)	-
Sex	Male	194 (85.5)	45 (88.2)	90 (84.1)	59 (85.5)	0.789
	Female	33 (14.5)	6 (11.8)	17 (15.9)	10 (14.5)	
Etiology of cirrhosis	Hepatitis B	100 (44.1)	25 (49.0)	48 (44.9)	27 (39.1)	0.958 ^a^
	Hepatitis C	41 (18.1)	8 (15.7)	19 (17.8)	14 (20.3)	
	Hepatitis B + D	23 (10.1)	4 (7.8)	12 (11.2)	7 (10.1)	
	NASH	30 (13.2)	6 (11.8)	15 (14.0)	9 (13.0)	
	Autoimmune	4 (1.8)	1 (2.0)	1 (0.9)	2 (2.9)	
	Alcohol-related	14 (6.2)	5 (9.8)	5 (4.7)	4 (5.8)	
	Cryptogenic	15 (6.6)	2 (3.9)	7 (6.5)	6 (8.7)	
Child–Pugh class	A	114 (50.2)	29 (56.9)	52 (48.6)	33 (47.8)	0.126
	B	90 (39.6)	18 (35.3)	48 (44.9)	24 (34.8)	
	C	23 (10.1)	4 (7.8)	7 (6.5)	12 (17.4)	
MELD-Na group	<15	142 (62.6)	31 (60.8)	69 (64.5)	42 (60.9)	0.260
	15–19	51 (22.5)	15 (29.4)	24 (22.4)	12 (17.4)	
	≥20	34 (15.0)	5 (9.8)	14 (13.1)	15 (21.7)	
Milan criteria (pretransplant)	Within	136 (59.9)	36 (70.6)	69 (64.5)	31 (44.9)	0.007
	Beyond	91 (40.1)	15 (29.4)	38 (35.5)	38 (55.1)	
UCSF criteria (pretransplant)	Within	158 (69.6)	41 (80.4)	80 (74.8)	37 (53.6)	0.002
	Beyond	69 (30.4)	10 (19.6)	27 (25.2)	32 (46.4)	
Bridging/downstaging	No	159 (70.0)	39 (76.5)	74 (69.2)	46 (66.7)	0.492
	Yes	68 (30.0)	12 (23.5)	33 (30.8)	23 (33.3)	
AFP (ng/mL)	<10	98 (43.2)	29 (56.9)	46 (43.0)	23 (33.3)	0.049 ^a^
	10–199	99 (43.6)	18 (35.3)	49 (45.8)	32 (46.4)	
	200–399	11 (4.8)	3 (5.9)	5 (4.7)	3 (4.3)	
	≥400	19 (8.4)	1 (2.0)	7 (6.5)	11 (15.9)	
Donor type	Living	194 (85.5)	43 (84.3)	91 (85.0)	60 (87.0)	0.908
	Deceased	33 (14.5)	8 (15.7)	16 (15.0)	9 (13.0)	
Cholangiocarcinoma component	No	203 (89.4)	47 (92.2)	99 (92.5)	57 (82.6)	0.087
	Yes	24 (10.6)	4 (7.8)	8 (7.5)	12 (17.4)	
Largest tumor diameter (mm, explant)	<20	39 (17.2)	10 (19.6)	19 (17.8)	10 (14.5)	<0.001 ^a^
	20–49	133 (58.6)	35 (68.6)	67 (62.6)	31 (44.9)	
	50–79	42 (18.5)	6 (11.8)	16 (15.0)	20 (29.0)	
	≥80	9 (4.0)	0 (0.0)	1 (0.9)	8 (11.6)	
	No viable tumor	4 (1.8)	0 (0.0)	4 (3.7)	0 (0.0)	
Number of tumors (explant)	1–2	148 (65.2)	35 (68.6)	70 (65.4)	43 (62.3)	0.148
	3–4	31 (13.7)	9 (17.6)	16 (15.0)	6 (8.7)	
	≥5	44 (19.4)	7 (13.7)	17 (15.9)	20 (29.0)	
	No viable tumor	4 (1.8)	0 (0.0)	4 (3.7)	0 (0.0)	
Milan criteria (explant)	Within	121 (53.3)	33 (64.7)	64 (59.8)	24 (34.8)	<0.001
	Beyond	106 (46.7)	18 (35.3)	43 (40.2)	45 (65.2)	
Microvascular invasion	No	163 (71.8)	46 (90.2)	82 (76.6)	35 (50.7)	<0.001
	Yes	64 (28.2)	5 (9.8)	25 (23.4)	34 (49.3)	
Macrovascular invasion	No	196 (86.3)	48 (94.1)	96 (89.7)	52 (75.4)	0.005
	Yes	31 (13.7)	3 (5.9)	11 (10.3)	17 (24.6)	
Histological grade	1	34 (15.0)	6 (11.8)	23 (21.5)	5 (7.2)	0.006
	2	126 (55.5)	32 (62.7)	59 (55.1)	35 (50.7)	
	3	63 (27.8)	13 (25.5)	21 (19.6)	29 (42.0)	
	No viable tumor	4 (1.8)	0 (0.0)	4 (3.7)	0 (0.0)	

Values are *n* (%) or median (IQR). *p* values: chi-square test for categorical and Kruskal–Wallis test for continuous variables. ^a^ More than 20% of cells had an expected count <5; interpret with caution. Tumor diameter and number are explant findings. Four patients without viable tumor on explant pathology after locoregional therapy are shown separately for grade, diameter, and number of tumors and were excluded from the corresponding *p* value calculations. Non-FDG-avid tumors were defined as those with a tumor-to-liver SUV ratio ≤1.00, that is, tumor FDG uptake not exceeding that of the background liver.

**Table 2:** Cumulative incidence of recurrence according to clinical and pathological characteristics.

Variable	Level	*n*	Recurrence	1-Year %	3-Year %	5-Year %	Gray’s Test *p*
Age	<60	139	24	8.2	14.3	16.8	0.337
	≥60	88	19	11.9	19.4	22.1	
Sex	Male	194	37	9.1	16.4	18.8	0.823
	Female	33	6	12.6	15.7	18.8	
Child–Pugh	A	114	24	14.5	17.3	20.2	0.660
	B	90	15	3.5	14.3	16.9	
	C	23	4	9.7	19.7	19.7	
MELD-Na	<15	142	33	13.8	19.0	23.0	0.009
	15–19	51	2	0.0	2.2	2.2	
	≥20	34	8	6.3	25.5	25.5	
Milan (pretransplant)	Within	136	15	3.8	6.1	10.2	<0.001
	Beyond	91	28	18.4	31.9	31.9	
UCSF (pretransplant)	Within	158	21	3.9	8.7	12.3	<0.001
	Beyond	69	22	22.5	33.7	33.7	
Bridging/downstaging	No	159	29	9.0	15.7	17.9	0.572
	Yes	68	14	11.0	17.5	21.0	
AFP (ng/mL)	<10	98	10	5.3	9.7	9.7	0.002
	10–199	99	23	10.5	17.0	22.7	
	200–399	11	2	9.1	18.2	18.2	
	≥400	19	8	28.0	46.7	46.7	
Donor type	Living	194	38	9.7	17.0	19.5	0.446
	Deceased	33	5	9.1	12.1	15.2	
Cholangiocarcinoma component	No	203	35	8.1	15.0	16.8	0.039
	Yes	24	8	22.4	27.0	36.2	
Largest diameter (mm)	<20	39	4	5.1	7.7	7.7	<0.001
	20–49	133	21	7.8	13.6	17.1	
	50–79	42	12	9.9	22.8	25.9	
	≥80	9	6	61.9	81.0	81.0	
	No viable tumor	4	0	0.0	0.0	0.0	
Total diameter (mm)	<20	25	1	4.0	4.0	4.0	<0.001
	20–49	102	13	5.0	10.2	12.4	
	50–99	59	12	7.2	12.8	19.5	
	≥100	37	17	31.6	49.7	49.7	
	No viable tumor	4	0	0.0	0.0	0.0	
Number of tumors	1–2	148	22	7.6	11.1	13.4	<0.001
	3–4	31	4	10.2	10.2	14.0	
	≥5	44	17	17.2	40.6	44.0	
	No viable tumor	4	0	0.0	0.0	0.0	
Microvascular invasion	No	163	25	7.0	12.2	14.3	0.012
	Yes	64	18	16.2	26.7	30.7	
Macrovascular invasion	No	196	32	8.0	13.5	15.9	0.014
	Yes	31	11	19.4	32.3	35.8	
Histological grade	1	34	3	5.9	5.9	5.9	<0.001
	2	126	17	5.8	11.0	14.8	
	3	63	23	20.0	33.9	35.7	
	No viable tumor	4	0	0.0	0.0	0.0	
SUV ratio category	≤1.00	51	2	2.1	4.4	4.4	<0.001
	1.01–1.43	107	10	4.7	5.7	8.8	
	>1.43	69	31	22.5	41.0	44.7	
SUV ratio (cutoff 1.43)	≤1.43	158	12	3.9	5.3	7.5	<0.001
	>1.43	69	31	22.5	41.0	44.7	

Values are cumulative incidences of recurrence (Aalen–Johansen estimator), with death without recurrence treated as a competing event; *p* values are from Gray’s test. Tumor diameter, number, invasion, and grade are explant findings; four patients without viable tumor on explant pathology after locoregional therapy are shown separately.

**Table 3:** Cox regression analyses for TTR and OS.

Variable	TTR Univariable HR (95% CI)	*p*	OS Univariable HR (95% CI)	*p*	TTR Multivariable HR (95% CI)	*p*	OS Multivariable HR (95% CI)	*p*
Age (per 10 years)	1.06 (0.73–1.54)	0.766	1.26 (0.96–1.66)	0.099	0.92 (0.62–1.36)	0.676	1.21 (0.92–1.60)	0.178
Female sex	1.00 (0.42–2.37)	0.998	1.14 (0.64–2.02)	0.652	—		—	
MELD-Na (per 5 points)	0.89 (0.66–1.20)	0.447	1.03 (0.85–1.25)	0.754	0.79 (0.59–1.05)	0.105	0.98 (0.81–1.19)	0.858
Child–Pugh B (vs. A)	0.80 (0.42–1.52)	0.487	1.15 (0.74–1.80)	0.533	—		—	
Child–Pugh C	0.94 (0.32–2.70)	0.902	1.03 (0.48–2.19)	0.945	—		—	
log_2_(AFP + 1)	1.24 (1.11–1.38)	<0.001	1.07 (0.98–1.17)	0.128	1.14 (1.01–1.27)	0.030	1.03 (0.94–1.13)	0.520
Beyond Milan (pretransplant)	3.29 (1.76–6.17)	<0.001	1.46 (0.95–2.23)	0.084	2.19 (1.10–4.35)	0.025	1.18 (0.75–1.85)	0.477
Bridging/downstaging	1.24 (0.66–2.35)	0.508	1.38 (0.88–2.15)	0.157	—		—	
Deceased donor	0.72 (0.28–1.84)	0.497	0.95 (0.53–1.72)	0.871	—		—	
Cholangiocarcinoma component	2.41 (1.12–5.21)	0.025	1.93 (1.07–3.50)	0.030	—		—	
Largest diameter (per 10 mm)	1.31 (1.17–1.45)	<0.001	1.11 (1.01–1.21)	0.025	—		—	
Number of tumors	1.24 (1.13–1.36)	<0.001	1.10 (1.02–1.19)	0.012	—		—	
Microvascular invasion	2.24 (1.22–4.13)	0.010	1.81 (1.15–2.85)	0.010	—		—	
Macrovascular invasion	2.81 (1.41–5.60)	0.003	2.82 (1.71–4.65)	<0.001	—		—	
Histological grade 3 (vs. 1–2)	3.70 (2.03–6.75)	<0.001	1.67 (1.07–2.61)	0.024	—		—	
log_2_(SUV ratio)	7.22 (4.14–12.59)	<0.001	3.01 (1.91–4.74)	<0.001	6.68 (3.58–12.47)	<0.001	2.79 (1.72–4.53)	<0.001
SUV ratio > 1.43	8.51 (4.34–16.70)	<0.001	2.91 (1.89–4.48)	<0.001	7.08 (3.53–14.18)	<0.001	2.76 (1.76–4.34)	<0.001

Multivariable model: SUV ratio, age, MELD-Na, log_2_(AFP + 1), and Milan status (base model). log_2_(SUV ratio) and SUV ratio >1.43 were entered in separate models with the same covariates; multivariable HRs of the other covariates are from the log_2_(SUV ratio) model. References: male sex for female sex, living donor for deceased donor.

## Data Availability

The data presented in this study are available upon reasonable request from the corresponding author. The data are not publicly available because of patient privacy and institutional restrictions.

## Supplementary Materials

The following supporting information can be downloaded at https://www.mdpi.com/article/10.3390/jcm15218611/s1, Table S1: Competing risk analysis of HCC recurrence; Table S2: Adjusted hazard ratios across SUV ratio cutoffs; Table S3: Incremental value of the SUV ratio over existing criteria (likelihood ratio tests); Table S4: SUV ratio and outcomes in subgroups; Table S5: Association between the SUV ratio and explant findings; Table S6: Sensitivity analyses; Table S7: Multivariable Cox regression analysis for recurrence-free survival; Figure S1: Association between the SUV ratio and recurrence modelled with a natural cubic spline.

## Abbreviations

AFP	Alpha-fetoprotein
AUC	Area under the curve
CI	Confidence interval
CT	Computed tomography
FDG	^18^F-fluorodeoxyglucose
HCC	Hepatocellular carcinoma
HR	Hazard ratio
IQR	Interquartile range
MELD-Na	Model for End-Stage Liver Disease–sodium
MRI	Magnetic resonance imaging
OR	Odds ratio
OS	Overall survival
PET	Positron emission tomography
RFS	Recurrence-free survival
ROC	Receiver operating characteristic
SUV	Standardized uptake value
TTR	Time to recurrence
UCSF	University of California, San Francisco
VOI	Volume of interest.
