# Supplementary material for: Beyond Morphology: Pretransplant Tumor-to-Liver FDG Uptake Ratio Predicts Hepatocellular Carcinoma Recurrence After Liver Transplantation
Source: J Clin Med. 2026 Nov 5;15(21):8611. doi: 10.3390/jcm15218611 (PMC13764036; doi:10.3390/jcm15218611)
Supplement: Supplementary file 1 [file jcm-15-08611-s001.zip › jcm-4641439-supplementary.pdf]

Supplementary Materials

Beyond Morphology: Pretransplant Tumor-to-Liver FDG Uptake Ratio Predicts Hepatocellular Carcinoma Recurrence after Liver Transplantation

Table S1. Competing risk analysis of HCC recurrence

Panel A. Cumulative incidence of recurrence

SUV ratio group	1 year, % (95% CI)	3 years, % (95% CI)	5 years, % (95% CI)
**Two groups**			
SUV ratio ≤1.43	3.9 (1.6–7.8)	5.3 (2.5–9.7)	7.5 (3.9–12.5)
SUV ratio >1.43	22.5 (13.3–33.2)	41.0 (29.0–52.6)	44.7 (32.1–56.5)
**Three groups**			
SUV ratio ≤1.00	2.1 (0.2–9.9)	4.4 (0.8–13.5)	4.4 (0.8–13.5)
SUV ratio 1.01–1.43	4.7 (1.7–10.0)	5.7 (2.3–11.3)	8.8 (4.3–15.3)
SUV ratio >1.43	22.5 (13.3–33.2)	41.0 (29.0–52.6)	44.7 (32.1–56.5)

Gray's test: $p < 0.001$ for both the two-group and three-group comparisons.

Panel B. Adjusted Fine–Gray analysis

Variable	Adjusted SHR (95% CI)	p value
SUV ratio (per doubling)	5.84 (3.31–10.29)	<0.001
SUV ratio >1.43 (vs. ≤1.43)	6.74 (3.41–13.32)	<0.001

Notes. Cumulative incidence was estimated using the Aalen–Johansen estimator, with death without recurrence treated as a competing event. Values in Panel A are percentages with 95% confidence intervals calculated using the log(−log) transformation. In Panel B, the continuous $\log_2$-transformed SUV ratio and the binary SUV ratio variable were evaluated in separate Fine–Gray models, each adjusted for age, MELD-Na, $\log_2$(AFP+1), and pretransplant Milan status. The SHR for the continuous SUV ratio represents a twofold increase in the ratio.
Abbreviations: AFP, alpha-fetoprotein; CI, confidence interval; HCC, hepatocellular carcinoma; MELD-Na, Model for End-Stage Liver Disease–sodium; SHR, subdistribution hazard ratio; SUV, standardized uptake value.

Table S2. Adjusted hazard ratios across SUV ratio cutoffs (base model).

Cutoff	n (>cutoff)	Recurrences (>cutoff)	TTR HR (95% CI)	p	OS HR (95% CI)	p
1.00	176	41	5.14 (1.23–21.50)	0.025	1.54 (0.86–2.78)	0.149
1.10	145	39	5.31 (1.87–15.07)	0.002	1.61 (0.99–2.62)	0.053
1.20	117	38	7.06 (2.73–18.25)	<0.001	1.87 (1.18–2.95)	0.008
1.30	92	35	6.93 (3.13–15.37)	<0.001	2.08 (1.33–3.25)	0.001
1.40	72	31	6.51 (3.25–13.07)	<0.001	2.52 (1.60–3.96)	<0.001
1.43	69	31	7.08 (3.53–14.18)	<0.001	2.76 (1.76–4.34)	<0.001
1.50	54	26	5.54 (2.90–10.59)	<0.001	2.49 (1.55–3.99)	<0.001
1.60	42	21	5.43 (2.87–10.29)	<0.001	2.93 (1.79–4.78)	<0.001
1.70	35	16	4.09 (2.14–7.83)	<0.001	2.63 (1.59–4.37)	<0.001
1.80	30	14	4.09 (2.08–8.05)	<0.001	2.64 (1.54–4.52)	<0.001
2.00	19	10	4.05 (1.87–8.77)	<0.001	2.16 (1.09–4.29)	0.027

All models adjusted for age, MELD-Na, $\log_2$(AFP+1), and Milan status.

Table S3. Incremental value of the SUV ratio over existing criteria (likelihood ratio tests).

Model	Outcome	−2LL (before)	−2LL (after)	LR χ^2	df	p
Milan + SUV	TTR	424.19	392.33	31.85	1	<0.001
UCSF + SUV	TTR	427.35	395.65	31.70	1	<0.001
Milan + AFP + SUV	TTR	416.15	387.65	28.50	1	<0.001
Base model (age, MELD-Na, AFP, Milan) + SUV	TTR	415.08	384.71	30.37	1	<0.001
RETREAT + SUV (explant)	TTR	409.24	387.01	22.23	1	<0.001
SUV + Milan + AFP (contribution of Milan/AFP)	TTR	400.10	387.65	12.45	2	0.002
Milan + SUV	OS	855.88	838.67	17.21	1	<0.001
UCSF + SUV	OS	857.09	839.47	17.62	1	<0.001
Milan + AFP + SUV	OS	854.48	838.35	16.14	1	<0.001
Base model (age, MELD-Na, AFP, Milan) + SUV	OS	851.38	836.41	14.97	1	<0.001
RETREAT + SUV (explant)	OS	845.26	834.11	11.15	1	<0.001
SUV + Milan + AFP (contribution of Milan/AFP)	OS	839.70	838.35	1.36	2	0.507

Likelihood ratio test: difference in −2 log likelihood between nested Cox models. The SUV ratio was entered as continuous $\log_2$-transformed variable. RETREAT is based on explant findings and is not available before transplantation.

Table S4. SUV ratio and outcomes in subgroups.

Subgroup	Outcome	n	Events	n (>1.43)	$\log_2$SUV HR (95% CI)	>1.43 HR (95% CI)	5-year estimate, % (≤1.43 / >1.43)
Within Milan (pre-transplant)	TTR	136	15	31	6.17 (1.94–19.59)	7.61 (2.67–21.66)	5.2 / 26.4
Within Milan (pre-transplant)	OS	136	47	31	2.68 (1.27–5.65)	3.46 (1.93–6.21)	77.3 / 45.2
Beyond Milan (pre-transplant)	TTR	91	28	38	5.86 (2.99–11.50)	6.78 (2.74–16.81)	12.0 / 60.3
Beyond Milan (pre-transplant)	OS	91	39	38	3.26 (1.73–6.14)	2.09 (1.10–3.97)	64.7 / 39.1
AFP <200	TTR	197	33	55	6.21 (3.23–11.95)	7.23 (3.48–15.02)	7.6 / 38.2
AFP <200	OS	197	73	55	2.64 (1.59–4.41)	2.71 (1.69–4.34)	72.5 / 46.6
Within Milan (ex-plant)	TTR	121	13	24	9.90 (1.92–51.23)	6.12 (2.04–18.32)	5.5 / 25.7
Within Milan (ex-plant)	OS	121	41	24	2.44 (0.92–6.44)	2.59 (1.33–5.05)	75.9 / 48.0

Hazard ratios were estimated using univariable Cox models within each subgroup. For TTR, values are cumulative incidences of recurrence with death without recurrence as a competing event; for OS, values are Kaplan-Meier survival estimates.

Table S5. Association between the SUV ratio and explant findings.

Outcome / model	Adjustment	Events	Effect estimate (95% CI)	p
Microvascular invasion	unadjusted	64	OR 4.54 (2.20–9.38)	<0.001
Microvascular invasion	adjusted (AFP, Milan)	64	OR 3.92 (1.87–8.21)	<0.001
Macrovascular invasion	unadjusted	31	OR 3.08 (1.41–6.70)	0.005
Macrovascular invasion	adjusted (AFP, Milan)	31	OR 2.83 (1.24–6.43)	0.013
Histological grade 3	unadjusted	63	OR 3.02 (1.53–5.96)	0.001
Histological grade 3	adjusted (AFP, Milan)	63	OR 2.35 (1.16–4.74)	0.017
Cox: base model	TTR		HR 6.68 (3.58–12.47)	<0.001
Cox: base model	OS		HR 2.79 (1.72–4.53)	<0.001
Cox: + MVI	TTR		HR 6.52 (3.41–12.45)	<0.001
Cox: + MVI	OS		HR 2.60 (1.58–4.30)	<0.001
Cox: + MVI + grade 3	TTR		HR 6.03 (3.12–11.67)	<0.001
Cox: + MVI + grade 3	OS		HR 2.52 (1.53–4.17)	<0.001
Cox: + MVI + grade 3 + macrovascular invasion	TTR		HR 5.79 (2.95–11.36)	<0.001
Cox: + MVI + grade 3 + macrovascular invasion	OS		HR 2.31 (1.38–3.86)	0.001

Table S6. Sensitivity analyses (HRs adjusted with the base model).

Analysis	Outcome	n	Events	log_2SUV HR (95% CI)	p	>1.43 HR (95% CI)	p
All patients	TTR	227	43	6.68 (3.58–12.47)	<0.001	7.08 (3.53–14.18)	<0.001
All patients	OS	227	86	2.79 (1.72–4.53)	<0.001	2.76 (1.76–4.34)	<0.001
SUV ratio of exactly 1.00 excluded	TTR	187	43	5.42 (2.80–10.48)	<0.001	5.43 (2.72–10.84)	<0.001
SUV ratio of exactly 1.00 excluded	OS	187	76	2.77 (1.63–4.71)	<0.001	2.64 (1.65–4.25)	<0.001
Non-FDG-avid (≤1.00) excluded	TTR	176	41	6.44 (3.25–12.78)	<0.001	6.03 (2.88–12.61)	<0.001
Non-FDG-avid (≤1.00) excluded	OS	176	72	3.16 (1.83–5.47)	<0.001	2.74 (1.68–4.46)	<0.001
Cholangiocarcinoma component excluded	TTR	203	35	6.06 (3.06–12.01)	<0.001	7.10 (3.31–15.23)	<0.001
Cholangiocarcinoma component excluded	OS	203	73	2.45 (1.46–4.12)	<0.001	2.51 (1.54–4.09)	<0.001
Cholangiocarcinoma component added to model	TTR	227	43	6.95 (3.69-13.10)	<0.001	7.06 (3.52-14.16)	<0.001
Cholangiocarcinoma component added to model	OS	227	86	2.87 (1.76-4.69)	<0.001	2.73 (1.73-4.29)	<0.001
Macrovascular invasion excluded	TTR	196	32	6.24 (2.96–13.16)	<0.001	7.53 (3.36–16.86)	<0.001
Macrovascular invasion excluded	OS	196	65	2.38 (1.31–4.31)	0.004	2.62 (1.55–4.43)	<0.001
No viable tumor in explant excluded	TTR	223	43	6.59 (3.53–12.29)	<0.001	6.95 (3.47–13.92)	<0.001
No viable tumor in explant excluded	OS	223	85	2.78 (1.72–4.50)	<0.001	2.71 (1.72–4.27)	<0.001
Bridging therapy excluded	TTR	159	29	10.07 (4.66–21.75)	<0.001	7.57 (3.22–17.78)	<0.001
Bridging therapy excluded	OS	159	56	2.85 (1.54–5.26)	<0.001	2.57 (1.46–4.54)	0.001
Bridging therapy added to model	TTR	227	43	6.61 (3.54–12.34)	<0.001	6.98 (3.48–13.97)	<0.001
Bridging therapy added to model	OS	227	86	2.74 (1.69–4.45)	<0.001	2.72 (1.73–4.28)	<0.001

All models adjusted for age, MELD-Na, log_2(AFP+1), and Milan status. In the model with the cholangiocarcinoma component added, the interaction between the SUV ratio and the cholangiocarcinoma component was not significant (log_2SUV: p=0.43 for TTR and p = 0.52 for OS; SUV ratio >1.43: p=0.76 for TTR and p=0.91 for OS).

Table S7. Multivariable Cox regression analysis for recurrence-free survival

Variable	Adjusted HR (95% CI)	p value
SUV ratio (per doubling)	2.92 (1.83–4.65)	<0.001
Age (per 10-year increase)	1.20 (0.91–1.59)	0.199
MELD-Na (per 5-point increase)	0.99 (0.81–1.19)	0.886
log_2(AFP+1) (per unit increase)	1.05 (0.96–1.14)	0.311
Beyond Milan criteria (vs. within)	1.26 (0.81–1.97)	0.303

Notes. The analysis included 227 patients and 89 recurrence-free survival events. Recurrence-free survival was defined as the time from liver transplantation to the first HCC recurrence or death from any cause, whichever occurred first. Patients who were alive without recurrence were

censored at their last follow-up. All five variables shown were included simultaneously in the model. The SUV ratio was log_2-transformed; its HR represents a twofold increase in the ratio. The HR for log_2(AFP+1) represents a twofold increase in AFP+1. Tied event times were handled using the Breslow method. Confidence intervals are Wald intervals; p values are two-sided.
Abbreviations: AFP, alpha-fetoprotein; CI, confidence interval; HCC, hepatocellular carcinoma; HR, hazard ratio; MELD-Na, Model for End-Stage Liver Disease–sodium; SUV, standardized uptake value.

Figure S1. Association between the SUV ratio and recurrence modelled with a natural cubic spline.

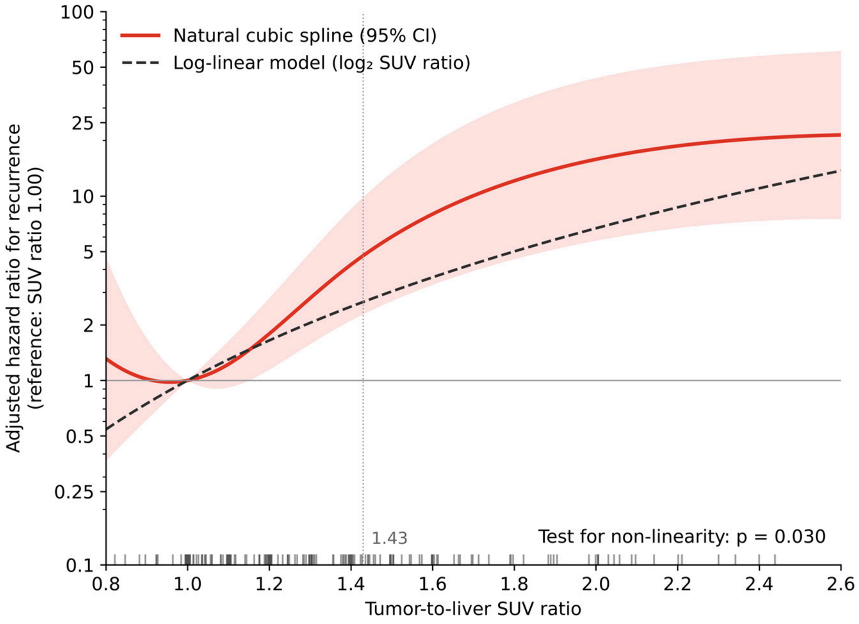

Figure S1. Adjusted hazard ratio for recurrence according to the tumor-to-liver SUV ratio, modelled with a natural cubic spline of log_2(SUV ratio) (three degrees of freedom) and adjusted for age, MELD-Na, log_2(AFP+1), and pretransplant Milan status. The shaded area indicates the 95% CI, the dashed line shows the log-linear model, and the reference is an SUV ratio of 1.00. Tick marks indicate individual observations.
